# Cell Therapy in Ischemic Heart Disease: Interventions That Modulate Cardiac Regeneration

**DOI:** 10.1155/2016/2171035

**Published:** 2016-01-05

**Authors:** Maximiliano I. Schaun, Bruna Eibel, Melissa Kristocheck, Grasiele Sausen, Luana Machado, Andreia Koche, Melissa M. Markoski

**Affiliations:** Laboratório de Cardiologia Molecular e Celular (LCMC), Instituto de Cardiologia/Fundação Universitária de Cardiologia, Porto Alegre, RS, Brazil

## Abstract

The incidence of severe ischemic heart disease caused by coronary obstruction has progressively increased. Alternative forms of treatment have been studied in an attempt to regenerate myocardial tissue, induce angiogenesis, and improve clinical conditions. In this context, cell therapy has emerged as a promising alternative using cells with regenerative potential, focusing on the release of paracrine and autocrine factors that contribute to cell survival, angiogenesis, and tissue remodeling. Evidence of the safety, feasibility, and potential effectiveness of cell therapy has emerged from several clinical trials using different lineages of adult stem cells. The clinical benefit, however, is not yet well established. In this review, we discuss the therapeutic potential of cell therapy in terms of regenerative and angiogenic capacity after myocardial ischemia. In addition, we addressed nonpharmacological interventions that may influence this therapeutic practice, such as diet and physical training. This review brings together current data on pharmacological and nonpharmacological approaches to improve cell homing and cardiac repair.

## 1. Introduction

Cardiovascular events are among the leading causes of morbidity and mortality worldwide [1], and ischemic heart disease (IHD), caused by coronary obstruction, accounts for 80% of deaths from cardiovascular disease [2]. Although current pharmacological and surgical therapies have promoted a decrease in mortality rates due to acute myocardial infarction (AMI), they cannot promote the recovery of the injured area. Many patients develop chronic complications related to ischemia or myocardial necrosis, such as congestive heart failure [3]. Therefore, there is a need to develop new strategies to promote coronary revascularization and restoration of cardiac function.

Cell therapy has emerged as a promising alternative strategy, since it involves the delivery of cells with regenerative potential, mainly through the release of paracrine and autocrine important factors that contribute to cell survival, angiogenesis, and tissue remodeling [4–6]. The different lineages of stem cells, which have shown therapeutic potential for cardiovascular disease, can be broadly classified as bone marrow derived cell (BMDC) [7], bone marrow derived mesenchymal stem cells (MSC) [8], adipose derived mesenchymal cell (ADSC) [9], hematopoietic stem cells (HSC) [10], and cardiac stem cells (CSC) [11].

Despite the progress made since the first clinical trial conducted by Menasché et al. [12], cell therapy is far from being an established treatment for patients with myocardial infarction. The lack of robust results due to the low rate of survival and poor retention of transplanted cells in the injured tissue [13] as well as the cell type and route of administration seem to affect the treatment success [14, 15].

In recent years, there has been a large effort to elucidate the mechanisms of stem cells in regenerating damaged tissues. One of the key mechanisms is the release of signaling molecules of injury and capture of the stem cells, which are involved in proliferation, migration, differentiation, and engraftment in the target tissue [16]. This process is called* cell homing* and it is characterized by a molecular axis resulting from the interaction of the chemokine Stromal-Derived Factor-1 (SDF-1 or CXCL-12) with its specific receptor, the CXC chemokine receptor type 4 (CXCR-4) [16]. This pathway is influenced by various cytokines that modulate the immune system and the expression of growth factors as well as other molecules activated in response to physiological and pathological tissue regeneration.

Homing, in its magnitude, can be influenced both by heart disease (especially ischemic) and by therapeutic process either positively or negatively. Often, drugs used in the treatment of diseases inhibit cellular processes and consequently cell proliferation necessary for the tissue repair. In contrast, nonpharmacological interventions such as diet and physical activity can promote adequate conditions for cell homing [36]. In this context, activation of homing is the first step for tissue regeneration. The objective of this review is to discuss the main mechanisms of cell therapy for regeneration and angiogenesis in myocardial ischemia, focusing on the factors that may influence this therapeutic practice, such as diet, physical training, and pharmacological interventions.

## 2. Pathological Factors Leading to Cardiac Remodeling

According to World Health Organization (WHO), in 2011, IHD was the leading cause of death worldwide [37]. The ischemic process is characterized by the lack of blood supply to the tissue due to an obstruction caused by a thrombus formed by fatty deposits or blood clots. The main cause of ischemia is hypoxia, which leads to a lack of glucose and oxygen supply to cells and consequently to cell death. The clinical outcome of atherosclerosis is AMI, characterized by cell death by necrosis due to a lack of blood supply [38]. According to Antman et al. [38], in most cases, myocardial infarctions are transmural; that is, the ischemic necrosis involves the entire or almost the entire thickness of the ventricular wall in the distribution of a single coronary artery. Subsequently, the subendocardial infarct is an ischemic necrosis area limited to a third or, at most, a half of the ventricular wall [39]. The consequent loss of cardiomyocytes in AMI reduces the ability of cardiac tissue to pump blood from the left ventricle, leading to a progressive ventricular dilatation and reduced ejection capacity [39]. Patients who survive myocardial infarction become predisposed to chronic complications arising from repair mechanisms. In response to myocardial injury, a sequence of molecular and physiological responses is triggered, leading to ventricular remodeling and heart failure [40].

Activation of the immune response by AMI is characterized by migration and aggregation of cells of innate immunity, activation of the complement cascade, secretion of cytokines, such as interleukin-1 (IL-1), interleukin-2 (IL-2), interleukin-6 (IL-6), and tumor necrosis factor type-alpha (TNF-*α*) [41], and increase in T and B cells [42] and ROS [43, 44]. This phenomenon is the main responsible for the progressive functional losses that characterize cardiac ischemic events. However, the high release of cytokines and growth factors and the expression of adhesion molecules are also essential for the recruitment of progenitor cells (such as tissue-resident cells) from the bone marrow and their circulation to the injured organ [45, 46]. This is characterized by the activation of a gradient of molecules and cells, which is also an evidence of the body's attempt to perform tissue repair.

Cell homing refers to this process whereby the cells are attracted to the chemoattractant gradient in response to ischemic tissue injury modulated by cytokines produced by damaged tissues or cells in apoptosis [47]. Progenitor cell homing is a physiological mechanism that occurs in all tissues for the replacement of cells. In this process, SDF-1 and its specific receptor, CXCR-4 [48], are the main molecules involved, allowing the adhesion and transmigration of cells, mainly circulating proangiogenic and hematopoietic stem cells [49]. In response to AMI, an increase in SDF-1 expression in the infarct and peri-infarct areas and a reduction in their levels to baseline values within 7 days have been demonstrated [50]. In AMI, SDF-1 also exerts its effects via activation of phosphatidylinositol 3-kinase (PI3K) and mitogen-activated protein kinase (MAPK) and hence activates the enzyme endothelial nitric oxide synthase (eNOS) and increases nitric oxide (NO) production [51]. Although the SDF-1/CXCR-4 axis is activated by physiological mechanisms, including the replacement of cells in apoptotic processes, therapeutic procedures can modulate activation of these progenitor cells. In this way, treatment with injection of stem cells (*cell therapy*) may have an important action on the activation of homing process.

Many therapeutic strategies have been investigated to reduce the adverse effects of heart disease and associated risk factors, such as ventricular remodeling and consequent chronic heart failure (CHF) [52]. Studies have shown that stem cell therapy has the potential to regenerate myocardial tissue, induce angiogenesis, and thus improve the clinical course of the disease [8, 53–55].  Table 1 shows the main clinical protocols, the cell source, the delivery process, and the outcomes in relation to the regenerative process in cell therapy.

## 3. Cell Therapy for Angiogenesis in Myocardial Ischemia

After the ischemic event, the restoration of blood flow and oxygen supply to the ischemic tissue results in the production of ROS, which are highly damaging to cells and can cause an exaggerated systemic inflammatory reaction [44, 56]. Thus, reperfusion, required for the recovery of cell function, can aggravate the injury of ischemia, causing irreversible damage to tissue and cell death. Both prolonged ischemia and reperfusion may cause myocardial hibernation, characterized by the occurrence of chronic ventricular dysfunction. The pathophysiology of hibernating myocardium seems to be complex, involving repetitive postischemic dysfunction, which leads to phenotypic changes in myocardial cells, degeneration of myocytes, and tissue fibrosis [57]. Additionally, the condition is associated with a dynamic inflammatory process characterized by expression of the monocyte chemoattractant protein-1 (MCP-1) and continuous infiltration of leukocytes [58]. Regarding this molecular context, cardiology is undergoing one of the biggest revolutions in decades: a series of preclinical and clinical studies supporting the hypothesis that the injured heart tissue could be repaired with stem cell administration and consequent formation of new vessels and myocytes [59]. Approaches in which the cells are used to produce factors promoting angiogenesis and regeneration of the ischemic tissue in the peri-infarct zone stand out [60].

Besides BMDC, MSC, ADSC, HSC, and CSC [23, 61], specific types of cells have been used for this purpose, such as those derived from skeletal myoblasts [12], induced pluripotent stem cells (iPSC) [62], and endothelial progenitor cells (EPC) [63]. These cells are used to stimulate angiogenesis by the expression of growth factors (vascular endothelial growth factor (VEGF), fibroblast growth factor, hepatocyte growth factor, granulocyte-colony stimulating factor, and angiopoietin-1 (Angpt-1)) [64–66] via a local paracrine mechanism in response to ischemia. These cells can also participate as precursor cells for vasculogenesis, acting as a vehicle to deliver therapeutic genes encoding angiogenic factors (VEGF, Angpt-1, and SDF-1/CXCR-4) and increasing the mobilization of EPC and subsequent neovascularization [65, 67].

In the scope of clinical cardiovascular research on neovascularization and cardiomyogenesis, considerable progress in the use of adult stem cells for cell transplantation has been made using HSC, BMDC, MSC, and EPC [60, 63]. Neovascularization in postmyocardial infarction patients can be mediated by the incorporation of vascular progenitor cells into the capillary or by the delivery of growth factors and cytokines that enhance angiogenesis by affecting mature endothelial cells [68, 69]. In this context, humoral and paracrine factors released from stem cells and progenitor cells can improve cardiac function by reducing the apoptosis of cardiomyocytes or by activating CSC to increase cardiomyogenesis [70]. In relation to the effectiveness of the use of adult stem cells for cell transplantation, the variability in the reported findings may be partly explained by differences in the delivery methods, treatment logistics, and target diseases [71]. Despite positive effects on recovery of cardiac function in most of the studied groups, no significant changes in the ejection fraction have been reported [17, 18, 20, 72, 73], showing the need for technical refinements, standardization of scientific protocols, and validation of the findings.

In addition, a reduction in the number and function of autologous stem cells associated with comorbidities has been reported. Also, the paracrine effect on “diseased cells” and how these cells can be regenerated before transplantation has been investigated. In this regard, some experimental studies have identified alternative strategies to enhance cell survival, retention, integration, and homing [74]. These include genetic modification of stem cells prior to transplantation (cell transduction with prosurvival genes) and pretreatment of cells with other molecules (e.g., P38 inhibitors and eNOS) [75, 76], aiming at promoting tissue angiogenesis. Besides, experimental studies have focused on the use of cardiomyocyte implantation over the long term and its functional integration in rodents, motivating the translation of this approach to a nonhuman primate model of myocardial lesion [77]. More recently, cardiac-derived stem cells originally postulated as cardiomyocyte progenitors have been used in clinical trials [11]. In these studies, these cells are obtained from myocardial biopsies and cultured for obtaining an autologous population [78]. Once the ischemic insult results in death of myocytes secondary to vascular damage and decreased regional blood flow, cell therapy seeks to promote angiogenesis and myogenesis in attempt to restore the physiological balance and to prevent the progression of heart failure. In light of current knowledge and the complexity of these mechanisms, the most effective treatment is positively influenced by the natural mechanisms of repair by the administration or local recruitment of cells capable of promoting angiogenesis and/or myogenesis [79]. Therefore, therapies for myocardial ischemia should target signaling molecules whose proregenerative properties are involved in therapeutic angiogenesis. Furthermore, the usual pharmacological treatments for cardiovascular diseases may also interfere in molecular pathways required for the success of cell therapy.

## 4. Pharmacological Effects on Cell Therapy

Advances in cardiovascular pharmacology in the last decades have been fundamental in reducing morbidity, mortality, and complications related to heart disease. IHD requires long term control medications. In clinical practice, the pharmacotherapy for IHD is based on the time of disease progression; in its acute stage, the myocardium undergoes reversible, morphological changes due to progressive injury represented by ischemic areas and necrosis and permanent damage in the chronic phase [80]. These stages are related to a variety of clinical presentations and potentially fatal complications requiring optimized pharmacological treatment.

However, the mechanism by which pharmacological intervention interacts with cell homing, survival, proliferation, and so on is poorly understood. Evidence shows that pharmacotherapy for the treatment of ischemic disease and underlying conditions may affect the success of cell therapy [26, 81]. Preclinical data indicate a dual effect of pharmacological intervention which is sometimes beneficial by improving the regenerative potential of the stem cell therapy [29] and is sometimes harmful to the repair process [30, 31].

Factors that complicate the maintenance, survival, and consequently the paracrine action of implanted cells, as well as their cardiomyogenic potential, constitute limitations of cell therapy and have been investigated in regenerative medicine [82]. Both* in vivo* and* in vitro* studies have shown that certain drugs can improve the quality of the local microenvironment and facilitate the survival and biological behavior of implanted cells [26, 81, 82]. Moreover, pharmacological treatment may influence the cardiomyogenic transdifferentiation capacity of transplanted cells, with functional improvements such as increased left ventricular ejection fraction (LVEF) [34]. All these properties may be applicable to and effective in clinical practice.

Previous studies have evaluated molecular and cellular aspects as well as signaling pathways of cardiac repair after treatment of stem cells [30, 34]. Different drugs used in clinical practice, including statins, aspirin, and beta-blockers, may act in signaling pathways involved in important cellular responses, including cell homing, activated by the SDF-1/CXCR-4 axis [29]. This can have a significant impact on the progress of cell therapy, particularly in regenerative medicine focused on cardiac repair.

### 4.1. Statins

As previously mentioned, the ischemic process may be caused by blockage of coronary arteries by atherosclerotic plaques. These plaques are prone to rupture and expose substances that induce thrombosis, causing AMI. Thus, an approach for treatment and prevention of AMI consists of reducing the risk factors for atherosclerotic disease. Statins, among other effects, inhibit atherogenesis by changing circulating levels of plasma lipoproteins and are currently used in patients with high risk of cardiovascular events [83]. In addition, experimental data have shown that statins may have beneficial effects on LVEF and postmyocardial infarction remodeling [84, 85].

The pharmacological treatment with statins also presents pleiotropic effects, specifically in inhibiting inflammatory, fibrotic, and apoptotic processes [86]. Such effects may lead to an improvement in the local microenvironment, which in turn enhances the survival capacity and functional recovery of implanted cells. Using an animal model of myocardial infarction, Yang et al. [26] showed promising results in using a combination of stem cells and statins in IHD. This synergism may be effective in regeneration and repair of normal myocardial function and postmyocardial infarction morphology.

In addition, experimental studies have confirmed the beneficial effect of atorvastatin on postinfarction [25, 27]. Animals treated with atorvastatin followed by MSC transplantation showed enhanced survival of implanted cells and significant improvement in cardiac function [25, 27]. The benefits of combination therapy (atorvastatin + MSC) can be at least partly mediated by increased expression and activation of the enzyme nitric oxide synthase (NOS), particularly the eNOS isoform [25]. Dong et al. demonstrated that atorvastatin leads to phosphorylation of protein kinase activated by adenosine monophosphate (AMPK), which results in the activation of eNOS [87].

Atorvastatin may also influence the signaling processes activated by the SDF-1/CXCR-4 axis [29]. Evidence indicates an increase of SDF-1 chemokine levels in animal models of myocardial infarction undergoing treatment with the drug, with consequent increase in CXCR-4 receptor activation, providing antiapoptotic and anti-inflammatory effects. These effects may be, at least in part, eNOS/NO dependent. Furthermore, activation of SDF-1/CXCR-4 axis culminates in the activation of a cascade of signaling pathways responsible for mobilization and stem cell graft which contribute to the improvement of cardiac function [29].

Furthermore, administration of statins in patients with coronary artery disease (CAD) may have an influence on endothelial function. As demonstrated by Vasa et al. [28] in a group of patients with CAD, atorvastatin treatment increased the serum levels of circulating EPC. Given the well-established role of EPC in the repair process after ischemic injury, these data suggest the potential of statins in improving neovascularization, which contributes to survival and maintenance of tissue viability in acute and chronic phases of myocardial ischemia, which may be beneficial for clinical cell therapy protocols.

### 4.2. Aspirin

Pharmacological therapy for the treatment or prevention of thrombosis or thromboembolism has been extensively used in clinical practice because of the high prevalence and serious consequences of these conditions, including AMI [88]. Antiplatelet agents are particularly important due to the essential role of platelets in the development of thrombosis. Aspirin belongs to the class of nonsteroidal anti-inflammatory drugs, but it is widely used in low doses in noninflammatory conditions, such as cardiovascular disorders, primarily because of its antiplatelet effect [88]. However,* in vitro* experimental evidence has demonstrated that aspirin exerts other effects on cell signaling mechanisms [30, 31]. This drug is of particular interest due to its ability to inhibit cell proliferation* in vitro* [89].

Aspirin can also induce cell apoptosis, mediated by mitochondrial caspase-3, which in turn requires the activation of Wnt/*β*-catenin [80, 81]. This signaling plays a critical role in self-renewal, differentiation, and survival of MSC [90]. In contrast to atorvastatin, the use of aspirin may also be associated with a decrease in the number of circulating EPC in humans, with a detrimental effect on neovascularization capacity. This effect may be dose- and time-dependent [32].

### 4.3. Beta-Blockers

Beta-blockers are prescribed to patients with myocardial infarction and/or hypertension. These drugs act in the blocking activity of *β*-adrenergic or *α*-adrenergic receptors [91]. Beta-blockers provide a decrease in heart rate and myocardial contractility and an increase in myocardial oxygen consumption, significantly reducing the overall workload of the heart and leading to the improvement of ischemic symptoms [92].

Carvedilol, in addition to the nonselective blockade of *β*1-adrenergic, *β*2-adrenergic, and *α*1-adrenergic receptors, presents unique antioxidant properties by promoting the removal of superoxide anions and hydroxyl radicals [93].* In vitro* studies demonstrate a protective effect of carvedilol against the deleterious effects of ROS induced by hydrogen peroxide and antiapoptotic effect on MSC [33]. Additionally, in AMI animal models, MSC transplantation in animals pretreated with carvedilol improved cardiac function, reduced fibrosis, increased angiogenesis and cardiomyocyte survival, and decreased caspase-3 expression in ischemic heart tissue. Hassan et al. [33] suggested that the decrease in apoptosis may be due to the synergism of the antioxidant properties of carvedilol and paracrine signaling of MSC, which ultimately can lead to a reduction of apoptosis of remaining cardiomyocytes in peri-infarct regions. The combined treatment may be a more promising strategy for cardiac regeneration clinical practice to potential patients for cell therapy who are often treated with carvedilol.

Tissue regeneration requires the presence of cells with preserved proliferative capacity. A recent study demonstrated that propranolol is able to interfere with the proliferation of normal human endothelial cells and human cancer cell line [94]. This effect occurs through the modulation of key regulators of cell cycle progression and changes in the state of activation of specific and essential proteins to the maintenance of the cytoskeletal dynamics.* In vitro* treatment of cells with antagonist drugs of beta-adrenergic receptors may affect VEGF signaling, which uses NO as a second messenger. In addition, propranolol is able to block VEGF-R2 phosphorylation, an important mitogenic regulator [94]. Data suggest that atenolol can also impair the vasculogenesis capacity of stem cells due to inhibition of NO generation of and proangiogenic growth factors, such as VEGF [95]. Vasculogenesis is strongly related to the signaling pathway mediated by VEGF.

Cardiac patients use beta-blocker drugs to reduce the cardiac workload and consequently reduce the energy consumption by the heart muscle. Cell transplantation may be impaired in these patients, with loss of proliferative, vasculogenetic, and angiogenetic efficiency by the implanted cells. Consequently, these cells lose their regenerative capacity, which defines the outcome of stem cell therapy for cardiac repair. Additionally, the stem cell microenvironment should also be considered, since beta-blockers drugs are commonly used continuously in order to maintain a desired plasma concentration for the expected therapeutic effect.

### 4.4. Other Drugs

Another reason for the limited effect of cell therapy for cardiac repair in clinical trials may be the low cardiomyogenic efficiency. Numasawa et al. [34] have investigated the* in vitro* and* in vivo* effect of angiotensin receptor blocker (ARB) drugs on cardiomyogenic capacity of human MSC derived from bone marrow. They found increased cardiomyogenic transdifferentiation efficiency* in vitro* and improved LVEF after infusion of pretreated MSC. In addition, immunohistochemical analysis revealed a significant increase in* in vivo *cardiomyogenesis. Examples of this class of drugs include losartan, valsartan, and candesartan, which act by preventing the action of angiotensin-II (Ang-II) (a potent vasoconstrictor agent) in its specific receptor in blood vessels [96]. Cells treated with ARB have shown greater* in vitro* transdifferentiation capacity, and transplantation of these cells promoted a significant improvement in cardiac function in AMI model [34].

The cardiomyogenic effect has also been observed with pioglitazone, a drug used to control blood glucose in diabetic patients whose risk factors are closely associated with the development of cardiovascular disease [35]. In this study, transplantation of cells pretreated with this drug improved the efficacy of* in vivo* cardiomyogenic transdifferentiation. Such effect possibly involved the activation of the nuclear receptor PPAR-*γ*, which regulates the expression of genes and affects the increase in cardiomyogenesis induced by pioglitazone.

The optimization of stem cell therapy for ischemic disease has been widely discussed. New evidence on how drug therapy and other interventions may affect cardiac repair could lead to new approaches to promote survival, maintenance, paracrine action, and cardiomyogenesis of the transplanted cells [82]. Understanding how cell therapy and pharmacotherapy interact could contribute to better targeting of cell therapy protocols, particularly those aimed at cardiac regeneration and repair.  Table 2 summarizes some of the drugs commonly used in clinical practice and their influence on tissue regeneration after the administration of adult stem cells for the treatment of cardiovascular diseases. Interestingly, nonpharmacological interventions, including diets and physical activity, can also modulate the regenerative process and influence the stem cell therapy outcome.

## 5. Nonpharmacological Interventions

When it comes to prevention of cardiovascular diseases, we must consider a healthy lifestyle since childhood, including a balanced diet and regular physical exercise [97]. Once heart disease is established, therapeutic interventions should be planned according to the phase (acute or chronic) of the disease. The benefits of the combination of diet and regular physical training (cardiac rehabilitation) are potentially higher soon after an ischemic event and include positive effects on myocardial oxygen demand, endothelial function, autonomic tone, coagulation and clotting factors, inflammatory and lipoprotein markers, and development of coronary collateral vessels [98]. In chronic phase, healthy diet and physical training help maintain the physiological functions of the body, blood pressure, oxygen expenditure, and functional capacity, by adjusting the limitations imposed by the cardiac event [99, 100]. In addition, it is well known that lack of exercise is among the major causes of chronic diseases, including the IHD. The main effects of diet and physical activity on cardiac regeneration are presented below.

### 5.1. Diet

In recent decades, the importance of diet and other lifestyle factors in the control of cardiovascular diseases has been strongly emphasized [99, 101]. Since 1960, several studies have focused on the search for dietary indicators of atherogenicity, such as energy and cholesterol saturated and unsaturated fatty acids content.

Numerous randomized trials have demonstrated that appropriate dietary interventions can decrease [101] or even prevent the occurrence of various chronic diseases [102–104], including heart diseases. When Keys et al. [105] began to study the Mediterranean region, they found that although the local population consumed a large amount of fat (35% to 40% of total daily calories), similar to Western countries, they had a low incidence of cardiovascular diseases. The authors described the “Mediterranean diet,” which was rich in vegetables (fruits, vegetables, greenery, breads, and whole grains), fish and chicken (occasional red meat), red wine (in moderation and at meal times), dairy products (yogurt and cheese mainly), and fats, represented by nuts and olive oil in abundance. These findings were the basis for the study by de Lorgeril et al. [106], a randomized clinical trial of secondary prevention, aimed at testing the influence of the Mediterranean diet on patients' evolution after AMI. The study had been planned to be conducted for five years but was stopped after 27 months due to the benefits observed in the experimental group, with 70% decrease in overall mortality, primarily by reduction of coronary mortality. Several evidences of the benefits of the Mediterranean diet in secondary prevention have been shown since then [107, 108].

In this way, beneficial dietary factors have also been used in protocols of stem cell therapy, since it is believed that adult stem cells, present in different tissues, play a regenerative role following an injury or insult [109, 110]. The challenge, however, is the low survival and differentiation of implanted cells, since most of these cells do not survive beyond 72 hours after transfusion [111]. Some studies have shown that this regeneration may be facilitated by a dietary adjuvant, such as the adoption of the Mediterranean diet.

Gorbunov et al. [111] treated CSC with resveratrol, a polyphenol with antioxidant and anti-inflammatory properties, and tested in a rat model left descending occlusion. The authors showed that the animals receiving the treated cells showed significant improvement in LVEF, fractional shortening, and cardiac output as compared to the control group after 1, 2, and 3 months of the procedure. The treated cells also had greater survival and engraftment, as well as increased expression of SDF-1 chemokine and important genes related to stress.

The intake of essential polyunsaturated fatty acids (PUFAs), as omega-3 (*ω*-3) fatty acids, was shown to reduce the cardiovascular risk by inducing the release of prostaglandins, leukotrienes, and thromboxanes [112]. Essential fatty acids are needed for the synthesis of prostaglandins and proteins and play an important role in cell regeneration, defense mechanisms, and tissue regeneration [113]. PUFAs also generate precursors of pro- and anti-inflammatory molecules that can interfere in the physiology of chronic diseases and inflammatory processes. Linolenic acid, for example, is important in the transport of fat [114], contributing to the maintenance of the epidermal barrier integrity and accelerating healing processes. It also acts in the modulation of cell membrane by protecting against injury and acting as an immunogenic and restorative agent for the tissue, promoting chemotaxis, angiogenesis, humid environment, and tissue granulation, and thereby promotes cellular nutrition [115].

In obesity and type 2 diabetes, accumulation of nonessential fatty acids affects the regeneration process of skeletal muscle [116]. This may be critical for obese and diabetic patients who suffered from muscle damage caused by strenuous physical activity and consequent local ischemia. Other studies have shown that certain vitamins and minerals such as vitamins B3, C, and D, folic acid, selenium, and retinoic acid may promote the proliferation and differentiation of stem cells [117], thereby enhancing stem cell homing. Wong et al. [118] showed that physiological concentration of vitamin D promotes vascular regeneration, which was associated with an increase in the number of angiogenic myeloid cells, despite increased plasma levels of SDF-1.

### 5.2. Physical Training

Sedentary lifestyle is an important public health problem that is directly related to increased cardiovascular risk, which can be reduced by regular physical exercise [119]. However, despite the extensive characterization of the adaptations induced by exercise training, such as capillary density, enzyme profiles, and contractile proteins [120–122], there is still a great need for research on the molecular events underlying the physiological responses to physical training.

In CHF, regular physical exercise is associated with improvement of peripheral metabolism [123], oxygen consumption, electrocardiographic parameters [124], and mortality rates [125]. Aerobic training can prevent the loss of endothelium-dependent vasodilation and recover its function in sedentary middle-aged individuals and in men over 60 years of age [126]. Aerobic training was also associated with beneficial changes in blood pressure, lipid metabolism, glucose metabolism, body weight, and shear stress and with improvements in antioxidant defenses [119].

Gunning et al. [127] found that 6 weeks of aerobic training increased myocardial perfusion in patients with IHD. Consecutive sessions of training can promote cytoprotection by increasing the expression of heat shock proteins, mainly HSP72, in heart tissue and reducing apoptotic markers in murine model of ischemia/reperfusion [128]. Even if performed late after AMI, evidences in rats have shown that exercise training reduces heart dilation and scar development occurring after AMI [129], possibly because of its association with a better perfusion of heart tissue.

Physical training has been shown to be efficient in increasing the number and caliber of arterial vessels both in skeletal muscle and in myocardium, particularly in the injured heart tissue [130]. The activation of proangiogenic factors (cytokines, vascular growth factors, and NO) stimulates the mobilization and migration of circulating EPC and induces tissue neovascularization [131]. The study by Eleuteri et al. [132] with CHF patients demonstrated a higher mobilization of EPC, higher serum levels of angiopoietin-2 (Angpt-2), and an improvement in brachial artery dilation after 3 months of aerobic physical training. Similarly, a study from our group [133] showed that 12 weeks of moderate intensity aerobic training (60–70% of *V*_O_2__max) was able to increase the flow-mediated dilation in brachial artery and the antioxidant response in healthy middle-aged subjects.

Even short duration physical training programs seem to be able to induce important endothelial adaptive responses. Concomitant with mobilization of EPC, there is a progressive rise in eNOS expression, an increase in neointimal formation, and a larger aortic diameter in mice trained for 28 days in running wheel [131]. In the study by Turan et al. [134], 3 weeks of aerobic training was able to increase the concentrations of CD34^+^/CD45^+^ and CD133^+^/CD45^+^ as well as the migratory capacity of EPC in patients at 14 days after AMI as compared to sedentary subjects. There was no difference in LVEF at rest between the groups. However, the increase of LVEF at peak stress was significantly higher after exercise training as compared to the control group.

Besides the direct influence on the endothelial function, physical training seems to optimize the regenerative capacity of the cardiac tissue. Nunes et al. [129] found that 8 weeks of aerobic training in a group of Wistar Kyoto rats after experimental induction of AMI improved the inflammatory and oxidative status by reducing the cardiac remodeling in animals with CHF. In patients with CHF, 8 weeks of aerobic training promoted an increase in LVEF, a higher mobilization of EPC, and an increase in SDF-1 expression, which induces angiogenesis and acts as a chemoattractant mediator for progenitor cells and represents the essential cytokine for cell homing [135]. Thus, in addition to cell mobilization, physical training is able to promote the regenerative potential of EPC. Furthermore, elderly individuals undergoing 12 weeks of physical training on a treadmill showed increased expression of the SDF-1 receptor CXCR-4 in EPC, which was associated with enhanced reendothelization capacity of these cells [136].

The regenerative response induced by physical training seems to be related to a modification in the inflammatory profile established by the AMI. When Wistar Kyoto rats with experimental AMI were submitted to 8 weeks of aerobic training on a treadmill, they showed reduced plasmatic levels of IL-6 and TNF-*α* and higher levels of the anti-inflammatory cytokine IL-10 as compared to sedentary animals [129]. The IL-6 levels were positively associated with heart weight, indicating the effect of the inflammatory response on cardiac hypertrophy after AMI. Also, in patients with CHF, physical training was able to reduce plasma levels of inflammatory markers related to endothelial dysfunction [137]. Patients who underwent cycle ergometer training for 12 weeks showed lower plasma levels of MCP-1, intercellular soluble adhesion molecule (ICAM), and vascular cell adhesion molecules (VCAM), which suggests an inhibition of vascular inflammation by physical training.

In addition, changes in tissue environment induced by physical training seem to contribute to the regenerative process after cardiac ischemia. Under oxygen deprivation, the expression of SDF-1 gene is regulated by the hypoxia inducible factor-1 (HIF-1), which leads to a selective expression of the HIF-1 protein in the ischemic heart in proportion to the decrease in oxygen pressure [138]. HIF-1 positively regulates the expression of SDF-1, which stimulates adhesion, migration, and homing of CXCR-4 positive progenitor cells in the ischemic tissue. In this context, it was demonstrated that 6 weeks of physical training on a treadmill was able to increase the expression of HIF-1 in heart tissue of Wistar Kyoto male rats [139]. This response to aerobic training mimics cell signaling triggered by the ischemic event of myocardial infarction.

AMI creates an inhospitable environment in cardiac tissue, characterized by increased ROS production, which in turn may be modulated by antioxidant responses induced by physical training. Six weeks of swimming was able to decrease superoxide radical levels and lipid oxidative damage and increase the expression of the antioxidant enzyme catalase (CAT) and the activity of glutathione peroxidase (GPx) in the left ventricle of male Wistar Kyoto rats [140]. These enzymes are specific for ROS, preventing significant changes in cellular REDOX state and functional impairment of the tissue [141].

Therefore, physical training may affect, in different ways, the cardiac tissue environment. This nonpharmacological intervention not only is involved in the prevention of cardiac ischemic events, mainly by maximizing endothelial function, but also plays a part in the regeneration of heart tissue after AMI. Cellular signaling in tissue repair and adaptive responses to physical training may minimize the functional losses resulting from ischemic damage. The systemic environment created by physical exercise is likely to be relevant for therapeutic approaches aiming at recovering cardiac function.

In nonpharmacological interventions, although positive effects on the recovery of cardiac function have been in the majority of treated groups, the improvement of LVEF (~15%) was not significant in all studies [72]. Apparently, the effects of physical training on IHD and CHF are related to the improvement in angiogenic responses, inflammatory profile, and functional capacity. Therefore, tissue regeneration including enhanced cell expression and adherence to target tissues provided by cell based therapies may be potentiated by these supporting approaches or by healthy behaviors [142].

## 6. Future Directions

In the last decade, the use of stem cells in cell therapy has been highlighted due to the capacity of these cells to promote tissue repair and enhance cell differentiation rate [143]. However, on long term, therapies using stem cells have shown low cell attachment to the graft, probably due to their low survival after injection into the patient. In contrast, the recovery of cardiac function is, in part, precisely related to the early action of these cells [144]. In this way, the need for new discoveries of molecular mechanisms to be applied in clinical practice dramatically increases. The low effectiveness of cell therapy in clinical trials with patients with heart disease [145, 146] may be attenuated by using these new findings in combination with gene therapy. A previous study from our group showed that the gene therapy provides continuous delivery of therapeutic proteins to the target site [147] and, at the same time, stem cells regulate the expression of growth factors by paracrine effect [66, 144]. Ong et al. [148] performed a combined delivery of CPC and plasmids carrying HIF-1 gene in mice after experimental AMI. The combined therapy resulted in prolonged survival of transplanted CPC, reduced infarct area, and increased vascularization.

The regenerative response greatly depends on paracrine factors that can activate quiescent stem cells and/or induce the proliferation of the existing cardiomyocytes [11]. Exosomes secreted by MSC have emerged as possible factors responsible for the beneficial effects of paracrine signaling after cell therapy. As proposed by Lai et al. [149], purified exosomes reduced the size of the infarcted area in mice, and exosomes secreted by MSC showed a cardioprotective component, considered as a mediator for tissue repair. The interaction of exosomal microRNA of the resident cells with the transplanted ones can promote the survival of cells in the target tissue [148]. In this context, a lot of studies used exogenous molecules introduced to stem cells, aiming at promoting their overexpression or inhibiting specific cell mechanisms. Interestingly cardiac microRNAs were found to regulate the expression of paracrine factors by stem cells [150] and the angiogenic process [151].

Another alternative to increase the number of transplanted stem cells in the target organ is the cell sheet therapy. Cell sheet is an innovative technology that aims to obtain aligned cells in the graft, maintaining their biological and mechanical properties [152]. Several cell types have been studied to obtain cell sheets composed of viable cells [153, 154]. Experimental* in vivo* studies that used mesenchymal stem cell sheet and fibroblast have demonstrated improvement in muscle contractility, increase in vasculogenesis, and decrease in cardiac muscle fibrosis [155, 156].

Despite advances in the development of techniques aimed at increasing the effectiveness of cell therapy, improvement of cell homing is still a challenge. It would be necessary to identify safe and effective strategies to deliver the cells to the damaged organ and, at the same time, to improve the adherence of these cells in the target site. Then, it is crucial to establish the best source of cells and dose of injection [157]. As discussed by Garbern and Lee [11], the understanding of different responses observed in experimental and human models of cardiac regeneration and related pathways is determinant for the scientific progress in the field of regenerative cardiology.

**Figure 1:**
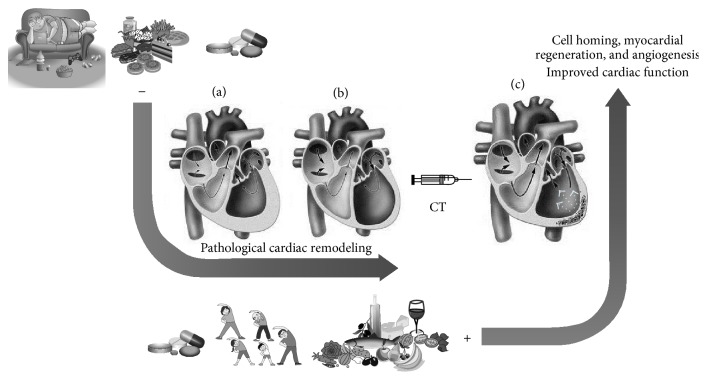
Factors that can modulate the cardiac regeneration in association with the stem cell therapy. The sedentary lifestyle and unhealthy dietary habits (as diets with elevated levels of fat and sugar) characterize risk factors that may contribute to the development of heart diseases, mainly due to the triggering of harmful issues that arise from the ischemic process which is generated in consequence of these behaviors. Therefore, the ischemia activates pathological cardiac remodeling mechanisms, the results of which may be the hypertrophy (a) or dilation (b) of the ventricular muscle, and both contribute to a decrease in ventricular ejection function. In an attempt to control the disease, there are commonly prescribed drugs that may interfere with mechanisms of cell proliferation and differentiation. These combined habits may adversely influence the effectiveness of cell therapy (CT). Subsequently, protocols that use stem cells are poorly effective in the regenerative process. Drugs that stimulate the antioxidant properties and control the inflammatory response, as well as the practice of physical activity and diets rich in cardioprotective elements (PUFAs, antioxidants, etc.), may balance the immune system. Besides that, a decrease in the ROS demand and increase of angiogenesis are stimulated by these approaches, providing a better environment for cell homing activation. This scenario intensifies mechanisms for cell regeneration and may lead to an increase in myocardial perfusion and improvement of the cardiac function (c). Thin black arrows indicate the blood flow in normal or damaged heart situation. White arrows suggest the improvement of the global contractility in the left ventricle after the regeneration process by stem cell enforcement (the figure was made from publicly available images).

In conclusion, many factors can positively or negatively influence and/or modulate the effectiveness of cell therapy in tissue recovery after damage in cardiovascular diseases, particularly in cardiac remodeling after ischemic injury (Figure 1). Cell therapy may have a significant role in the activation of homing mechanism, and therapeutic approaches are capable of activating progenitor cells, thereby improving heart tissue repair and ventricular remodeling. Current knowledge indicates that the most effective treatment available is positively influenced by the natural mechanisms of repair through the administration or local recruitment of cells capable of promoting angiogenesis and/or myogenesis. In this context, therapies for myocardial ischemia should target signaling molecules involved in angiogenesis. Besides, usual pharmacological treatments for cardiovascular diseases may also affect molecular pathways related to the success of cell therapy. The understanding of how cell therapy and pharmacotherapy interact could contribute to better targeting of clinical protocols. In addition to traditional pharmacological treatments, nonpharmacological interventions, such as diet and physical exercise, seem to be able to promote angiogenesis and generate a favorable environment for cell mobilization, survival, and repair.

## Figures and Tables

**Table 1:** Clinical trials using cell therapy protocols for ischemic heart disease.

Year, acronym	Disease	Cell source	Route of delivery	Primary outcome	Reference
2004	AMI (*n* = 69)	MSC, 18 days after AMI (9 × 10^9^)	Intracoronary	6 months after therapy ↑ LVEF (*p* = 0.01)	Chen et al. [8]

2004, BOOST	AMI (*n* = 60)	BMMC, 5 days after AMI	Intracoronary	6 months after therapy ↑ LVEF (*p* = 0.0026)	Wollert et al. [17]

2005	AMI (*n* = 19)	CD133^+^ cells, 12 days after AMI (15 × 10^6^)	Intracoronary	4 months after therapy ↑ LVEF (*p* = 0.005)	Bartunek et al. [18]

2005, MYSTAR	AMI (*n* = 60)	BMSC, early group, 3–6 weeks, and late group, 3-4 months after AMI (2 × 10^8^ intramyocardial; 1,3 × 10^9^ intracoronary)	Intramyocardial/intracoronary	3 months after therapy ↑ LVEF (*p* = 0.039, early group) ↓ infarct size (*p* = 0.001, early group)	Gyöngyösi et al. [19]

2006	AMI (*n* = 67)	BMSC, 1 day after AMI (2 × 10^8^)	Intracoronary	4 months after therapy *↔* LVEF (*p* = 0.36) ↓ infarct size (*p* = 0.036)	Janssens et al. [20]

2008, MAGIC	Left ventricular dysfunction/AMI	Autologous skeletal myoblasts (4–8 × 10^8^)	Intramyocardial	6 months after therapy *↔* LVEF (*p* = 0.66) *↔* LVEDV (*p* = 0.22) *↔* LVESV (*p* = 0.79)	Menasché et al. [12]

2012 (meta-analysis)	AMI or ischemic heart disease (50 studies; *n* = 2625)	BMDC	Intramyocardial or intracoronary	↑ LVEF (*p* < 0.0001) ↓ infarct size (*p* < 0.0001) ↓ LVESV (*p* < 0.0001) ↓ LVEDV (*p* < 0.0001)	Jeevanantham et al. [21]

2012, POSEIDON	Ischemic cardiomyopathy (*n* = 30)	MSC (2 × 10^7^, 1 × 10^8^, 2 × 10^8^)	Transendocardial	13 months after therapy ↓ infarct size (*p* = 0.001) *↔* LVEF (*p* = 0.11)	Hare et al. [22]

2013, C-CURE	CHF/recent AMI (*n* = 48)	Cardiopoietic stem cells (6–12 × 10^8^)	Intramyocardial	6 months after therapy ↑ LVEF (*p* < 0.0001) ↓ LVESV (*p* < 0.0001)	Bartunek et al. [23]

2014, PRECISE	Ischemic cardiomyopathy/CHF (*n* = 27)	ADSC (42 × 10^6^)	Transendocardial	6 months after therapy *↔* infarct size (*p* = 0.5) *↔* LVEF (*p* = 0.8)	Perin et al. [9]

2014	Ischemic heart failure (*n* = 39)	BMMC (8.4 × 10^8^)	Intramyocardial	1 year after therapy *↔* LVEF (*p* = 0.59) ↓ infarct size (*p* = 0.0002)	Pätilä et al. [24]

AMI, acute myocardial infarction; BMMC, bone marrow mononuclear cells; MSC, mesenchymal stem cells; ADSC, adipose derived stem cells; LVEF, left ventricular ejection fraction; LVEDV, left ventricular end-diastolic volume; LVESV, left ventricular end-systolic volume; BMSC, bone marrow derived stem cells; CHF, chronic heart failure.

**Table 2:** Pharmacological intervention and the interference in the tissue regeneration process after use of adult stem cells for treatment of cardiovascular diseases.

Drug	Therapeutic class	Pharmacological action	Interference in regenerative process	Reference
Simvastatin	Statin	Antidyslipidemic and antiatherosclerotic	Improvement of local microenvironment, promotes cell survival (*in vivo*)	Yang et al. [25] Yang et al. [26] Cai et al. [27]

Atorvastatin	Statin	Antidyslipidemic and antiatherosclerotic	Increase in circulating EPC (humans), neovascularization	Vasa et al. [28]
			Antiapoptotic effect (*in vivo*)	Qiu et al. [29]

Aspirin	Nonsteroidal anti-inflammatory	Antiplatelet agent	Inhibits cell proliferation (*in vitro*), induces apoptosis Lowers circulating EPC level (humans)	Wang et al. [30] Deng et al. [31] Lou et al. [32]

Carvedilol	Beta-blocker	Antihypertensive, other actions	Antiapoptotic and antioxidant effects (*in vitro* and *in vivo*)	Hassan et al. [33]

Candesartan	Angiotensin receptor blocker	Potent vasoconstrictor	Increases cardiomyogenic transdifferentiation (*in vitro* and *in vivo*)	Numasawa et al. [34]

Pioglitazone	Antidiabetic	Glycemic control	Increases cardiomyogenic transdifferentiation (*in vivo*)	Shinmura et al. [35]

EPC, endothelial progenitor cell.
